# The intention to leave among academics in Iran: an examination of their work-life quality and satisfaction

**DOI:** 10.1186/s12912-024-01720-7

**Published:** 2024-01-15

**Authors:** Akram Ghahramanian, Farzaneh Bagheriyeh, Parvaneh Aghajari, Mohammad Asghari-Jafarabadi, Pedram Abolfathpour, Azad Rahmani, Amirreza Nabighadim, Alireza Hajieskandar

**Affiliations:** 1 The National Agency for Strategic Research in Medical Education, Tehran, Iran; 2 Department of Medical-Surgical Nursing, School of Nursing and Midwifery, Tabriz University of Medical Sciences, Tabriz, Iran; 3 Department of Medical-Surgical Nursing, School of Nursing and Midwifery, Urmia University of Medical Sciences, Urmia, Iran; 4 Department of Pediatric Nursing, School of Nursing and Midwifery, Maragheh University of Medical Sciences, Maragheh, Iran; 5 Cabrini Research, Cabrini Health, Malvern, VIC 3144 Australia; 6 Biostatistics Unit, School of Public Health and Preventative Medicine, Faculty of Medicine, Nursing and Health Sciences, Monash University, Melbourne, VIC 3004 Australia; 7 Department of Psychiatry, School of Clinical Sciences, Faculty of Medicine, Nursing and Health Sciences, Monash University, Clayton, VIC 3168 Australia; 8 Road Traffic Injury Research Center, Tabriz University of Medical Sciences, Tabriz, Iran; 9 Student Research Committee, School of Nursing and Midwifery, Urmia University of Medical Sciences, Urmia, Iran; 10 Pediatric Urology and Regenerative Medicine Research Center, Children’Medical Center, Tehran University of Medical Sciences, Tehran, Iran; 11 Department of Computer, Bonab Branch, Islamic Azad University, Bonab, Iran

**Keywords:** Academic, Intention to leave, Satisfaction, Worklife

## Abstract

**Background:**

Despite the importance of faculty retention, there is little understanding of how demographic variables, professional and institutional work-life issues, and satisfaction interact to explain faculty intentions to leave. This study aimed to investigate the intention to leave among academics and their Work-Life Quality and Satisfaction.

**Methods:**

This is a descriptive cross-sectional study conducted by 8 faculties affiliated to Urmia University of Medical Sciences located in Urmia, West Azarbaijan province, Iran. The participants in the study were 120 faculty members from Nursing and Midwifery, Medicine, Allied health professions, and Health management and medical information faculties. The Work-Life Quality and Satisfaction scale, and the intension to leave scale were used for data collection. Uni- and multivariable linear regression analyses were employed to determine predictors of the intention to leave (*P*-values < 0.05).

**Results:**

The mean scores of all dimensions of Work-Life Quality and Satisfaction scale, and intention to leave were in an average level. There is a negative correlation between Work-Life Quality and Satisfaction subscales, along with demographic factors, and the intention to leave (*P* < 0.05), while multivariate analysis showed that work experience and Discipline were significant independent predictors of intention to leave (*P* < 0.05).

**Conclusions:**

In order to improve education in universities, it is necessary to pay attention to the conditions of creating job satisfaction in academics. Considering the high intention to leave among Nursing lecturers, without sufficient support of nursing schools in terms of human resources, it may suffer by the lack of academic staff; eventually the quality of education will reduce in undergraduate nursing in the long term.

## Background

The role of educational institutions in the development of human capital is crucial for the progress of any nation [1]. The academic staff within a university plays a vital role, and the number, quality, and effectiveness of faculty members greatly impact the quality of education provided [2]. It is widely recognized that the success of any organization is closely tied to the abilities and contributions of its employees [3].

The employment landscape in the education sector has become increasingly competitive, with institutions striving to maintain their reputation and gain a strategic advantage [4]. With the rise in job opportunities in higher education, retaining competent faculty members has become essential. Failing to retain employees can have severe repercussions for an organization [5].

Employee turnover has become a chronic issue across different types and sizes of organizations, and numerous studies emphasize the importance of retaining talented individuals [6]. In the field of education, replacing human capital, particularly in universities, is an expensive endeavor. Therefore, universities and governments must promptly and earnestly address talent turnover [7].

While satisfaction is a well-studied concept, colleges continue to face the challenge of motivating and satisfying their faculty members [8, 9]. Career motivation is considered one of the key individual factors impacting the quality of work life. Therefore, it is important to improve the quality of work life by fostering an environment that respects employees, encourages their active participation in decision-making processes, addresses their needs, and seeks to build trust with officials [10]. Fewer studies have focused on faculty members of medical sciences, who often encounter issues such as overcrowded classrooms, time pressure, and increased workload [11, 12].

Academic faculty members are considered national assets and understanding their intention to leave their positions is of utmost importance. The departure of experienced faculty members poses serious problems for universities, particularly regarding the quality of educational and research services they provide [13]. In today's academic landscape, faculty members bear significant responsibilities in education, research, therapeutic services, executive activities, and personal development [14]. Balancing multiple roles within a university, alongside external pressures from both the organization and the community, can significantly influence their perceived work-life balance satisfaction. This, in turn, impacts their job satisfaction and their intention to leave the organization [15].

On one hand, faculty satisfaction relies on the levels of satisfaction experienced by students, colleagues, and administrators [16]. On the other hand, Weale et al. (2019) suggested that faculty satisfaction is influenced by both work and non-work aspects of their lives [17]. Job satisfaction is a critical factor in motivating faculty members, as it reflects their personal contentment and fulfillment within their roles [12].

Building on the previous work of Kalkins et al., (2019) satisfaction levels strongly predict the intention of faculty members to leave academia. Additionally, the intent to leave one's position is a significant predictor of the intent to leave one's institution [18]. Turnover intention refers to an employee's intent to voluntarily leave their job or organization [19, 20]. Voluntary turnover is associated with decreased individual performance and increased costs for organizations [11].

Johnsrud (1996) proposed that faculty work-life can be influenced by their professional priorities, perceived institutional support, and erosion of quality of life over the course of their careers. Addressing these factors can improve the overall climate and culture within academic institutions [21], ultimately impacting faculty morale and their likelihood of leaving their positions or careers [22].

Multiple workplace roles undertaken by university academics, coupled with pressures from the organization and the community, are often considered significant factors that impact their perceived work-life balance satisfaction. This, in turn, influences their overall occupational attitudes, including job satisfaction, organizational commitment, and their intention to leave the organization [23].

Relatively few studies have examined the intent or inclination of faculty members in the field of Medical Sciences to leave their current positions [24, 25]. Some studies indirectly address this issue by exploring the faculty's intent to stay, either at their current institution or within public colleges in general [25, 26].

Despite the fundamental importance of faculty retention, there is limited understanding of the factors related to satisfaction, professional work life, and institutional work life that can explain faculty members' intentions to leave at a national level. As a result, this study aimed to investigate the intention to leave among academics and their work-life quality and satisfaction to response the following questions:

1. What are the academic members' perceptions of work- life quality and satisfaction?
2. What is the role of various individual, social, and occupational characteristics on academic members' perceptions of work- life quality and satisfaction?
3. What is the role of various individual, social, and occupational characteristics; and work- life quality and satisfaction in shaping faculty members' intentions to leave.

## Methods

### Design and participants

The current study is a cross-sectional descriptive study. All faculties affiliated to Urmia University of Medical Sciences, including Nursing and Midwifery, Health, Medicine, Dentistry, Pharmacy, and Health Management & Medical Information were included in the study. From these faculties, faculty members who had at least one years of teaching experience at the university; at least a master's degree; and were willing to complete the study instruments were enrolled. Those who worked part-time, or hourly were not included in the study. From March to June 2022, eligible faculty members were approached to participate in the study. The sample size (*n* = 115) was calculated in G power based on point biserial correlations between main outcomes satisfaction and intention to leave, considering a power = 0.80, an α = 0.05 based on the amount of correlation reported in a similar study [27]. Considering 20% attrition rate, the sample size increased to at least 138 participants.

The participants were selected through stratified random sampling considering a sample size in strata proportionate to the number of faculty members in the colleges.

### Data collection

#### Survey instruments

The data collection instruments included three main parts: first part with socio-demographic characteristics including age, sex, degree, grade, full timing, discipline, and teaching hours in Undergraduate, Master's, and PhD's teaching. Questionnaires that were incomplete by 10% or more were excluded from the study. Among the 145 questionnaires that were distributed and after discarding the distorted or incomplete questionnaires, 120 questionnaires were valid and the response rate in this study was 82.75%.

### Work-life quality and satisfaction scale

Work-life quality and satisfaction scale used in this study was developed by Rosser in an institutional climate study [27, 28]. The Rosser used the National Study of Postsecondary Faculty (NSOPF) database related to a survey sponsored by the National Center for Educational Statistics and the National Science Foundation to measure the various issues and topics concerning the quality of faculty members’ professional and institutional work-life in higher education institutions. He conceptualized the individual-level perceptions of faculty members’ work-life quality and satisfaction on their intent to leave [27]. Work-life quality and satisfaction scale is designed with 23 items in two sections and measures two related constructs including work-life quality and satisfaction. The items are measured on a 6-point scale (1 – Strongly Disagree, 2 – Disagree, 3 – Slightly Disagree, 4 – Slightly Agree, 5 – Agree, and 6 – Strongly Agree). The work-life quality section was measured by three dimensions including the professional development (alpha = 0.87), administrative support (alpha = 0.91), and technology support (alpha = 0.88). Respondents were asked to indicate from 1–6 score, indicating poor to excellent, statements regarding the quality of their professional and institutional work-life [27].

The satisfaction section in this scale was measured by three interrelated dimensions including the advising and course workload, benefits and security, and overall satisfaction. The first dimension with five statements was satisfaction with advising and course workload (alpha = 0.97). The second dimension with six items focused on their benefits and security (alpha = 0.76). Faculty members were also asked to self-report their overall level of satisfaction on a scale of 1–6, with 1 indicating very dissatisfied and 6 indicating very satisfied [27].

### Intention to leave

The intention to leave scale used in this study was designed by Rosser and Johnsrud in a study that aimed to conceptualize the effect of work environment variables and morale on the intention to leave [22]. The scale consists of four items, which ask faculty the likelihood to which they will leave their current position, their current institution, the teaching profession, and higher education. Items were measured on a 6-point scale (1–Highly Unlikely, 2–Unlikely, 3–Somewhat unlikely, 4–Somewhat likely, 5–Likely, and 6–Highly likely), where higher scores reflect individuals who possess a greater intent to leave.

### Statistical analyses

IBM SPSS Statistics software (version 20) (IBM SPSS Statistics, IBM Corp, Armonk, USA) was used to analyze the data at an alpha level of 0.05. Socio-demographic characteristics were summarized using frequency (percentage) and mean (standard deviation) for categorical and numeric variables, respectively. Independent t-test, one-way ANOVA analysis followed by Tukey post hoc tests, and Pearson's correlation were used to investigate the difference across the demographic characteristics for intension to leave scores. Uni- and multivariable linear regression analyses were employed to determine predictors of the intention to leave, with variables found to be significant in the univariable model (*P*-values < 0.05) included as independent variables in the multivariate model. Categorical variables were coded into dummy variables prior to regression analysis. Assessment of skewness (within ± 1.5) and kurtosis (within ± 2) indicated that the intention to leave data adhered to a normal distribution. The validity of the regression analysis was ensured by verifying assumptions, including the normality of residuals, homoscedasticity, and linearity of the variable relationships, which were confirmed [29].

### Ethical considerations

The present study was approved by the National Agency for Strategic Research in Medical Education, Tehran, Iran (code: 990,295). The study followed accepted ethical standards, as outlined in the Declaration of Helsinki. Participants were given detailed explanation on the study purpose, voluntary participation, and offered a written informed consent to obtain signature before presenting the study self-reporting questionnaires to be completed.

## Results

Table 1 presents the categorical socio-demographic characteristics and their association with work-life quality and satisfaction, and intention to leave. The results indicated that married faculties (M = 3.70, SD = 0.50) had a slightly higher work-life quality and satisfaction than single faculties (M = 3.47, SD = 0.43). There was no significant difference between subjects according to their gender, degree, or involvement in education. However, faculties who more involved in clinical teaching (M = 3.56, SD = 0.42) were significantly less satisfied with work-life quality than those who were less involved (M = 3.83, SD = 0.58).

**Table 1:** Work-related characteristics of the faculty members and their relationship with the work-life quality and satisfaction, and intention to leave (*n* = 120)

Variables	Sub-group	Frequency (percent)	Work-life quality and satisfaction scale	Intention to leave scale
Gender	Male	72 (60)	3.63 (.52)	2.78 (1.22)
	Female	48(40)	3.69 (.45)	2.66 (1.48)
Marital Status	Married	98(81.7)	3.70 (.50)^*^	2.67 (1.29)
	Unmarried	22(18.3)	3.47 (.43)	3.01(1.50)
Degree	Master	17(14.2)	3.63 (.46)	3.01 (1.88)
	Ph.D	103(85.8)	3.66 (.50)	2.68 (1.22)
Grade	Instructor	19(15.8)	3.67 (.59)	2.88 (1.88)
	Assistant Professor	60(50)	3.66 (.51)	2.77 (1.29)
	Associate Professor	26(21.7)	3.53 (.45)	2.77 (1.16)
	Professor	15(12.5)	3.86 (.34)	2.30 (.92)
Undergraduate education	Yes	108(90.0)	3.67 (.50)	2.74 (1.37)
	No	12(10.0)	3.54 (.46)	2.64 (.90)
Master’s education	Yes	91(75.8)	3.69 (.48)	2.62 (1.29)
	No	29(24.2)	3.57 (.53)	3.08 (1.41)
PhD’s education	Yes	42(35.0)	3.56 (.55)	2.60 (1.17)
	No	78(65.0)	3.71 (.46)	2.80 (1.41)
Clinical education	Yes	78(65.0)	3.56 (.42)	2.80 (1.40)
	No	42(35.0)	3.83 (.58)*	2.60 (1.18)
Full time	Yes	108(90)	3.67 (.51)	2.65 (1.27)
	No	12(10)	3.58 (.35)	3.41 (1.65)
Administrative position	Yes	28(23.3)	3.75 (.48)	2.57 (1.25)
	No	92(76.7)	3.63 (.50)	2.78 (1.35)
Discipline	Medicine	35(29.2)	3.66 (.61)	2.54 (.98)
	Nursing	25(20.8)	3.66 (.36)	3.44 (1.55)*
	Midwifery	14(11.7)	3.59 (.21)	1.89 (1.00)
	Allied health professions	36(30.0)	3.67 (.55)	2.70 (1.37)
	Health management and medical information	10(8.3)	3.71 (.46)	2.90 (1.38)
		**Mean (SD)**	**Correlation coefficient with** Work-life quality and **satisfaction**	**Correlation coefficient with intention to leave**
Age (Years)		43.18 (8.20)	0.11	-.31^**^
Fulltime duration (Years)		10.86 (7.89)	0.08	-.20^**^
Work experience (years)		12.30 (8.77)	0.1	-.31^**^
Clinical education (hours in week)		23.50 (16.31)	0.04	0.08
Research (hours in week)		18.84 (14.03)	-.21^**^	.18^**^
Income		22.50 (6.52)	0.13	-0.15
Children		1.40 (.65)	0.03	0.01
Teaching at undergraduate in semester (credit)		8.83 (4.52)	-.19^*^	0.18
Teaching at master in semester (credit)		4.15 (2.28)	0.18	-0.03
Teaching at Ph.D. in semester (credit)		6.26 (4.23)	0.08	0.06
Classroom education (hours in week)		11.54 (5.71)	0.08	-0.12
Practical/laboratory education (hours in week)		5.43 (5.23)	0.01	-0.11
Thesis/dissertation work (hours in week)		8.32 (5.90)	-0.03	-0.05
Organizational affairs(hours)		11.87 (11.29)	0.03	-0.03

^*^P< 0.05, significantly different from other categories

^**^P< 0.05, significant correlation

There was no significant relationship between being full time and having an administrative position with the work-life quality and satisfaction of faculty members. Regarding the discipline, there was no difference between different disciplines in terms of work-life quality and satisfaction. However, faculties from Midwifery (M = 3.59, SD = 0.21), Medicine (M = 3.66, SD = 0.61), and Nursing (M = 3.66, SD = 0.36) disciplines had lower work-life quality and satisfaction compared to Allied health professions (M = 3.67, SD = 0.55) and Health management and medical information (M = 3.71, SD = 0.46) disciplines. Table 1 also shows that nursing faculty members (M = 3.44, SD = 1.55) had a relatively higher intention to leave than faculty members of other disciplines. The rest of the categorical socio-demographic variables did not show a significant relationship with the intention to leave.

Besides, the results indicated that there was an inverse relationship between research hours per week (*r* = -0.21, *p* < 0.05) and teaching at undergraduate level (*r* = -0.19, *p* < 0.05) with work-life quality and satisfaction. Also, the variables of age (*r* = -0.31, *p* < 0.05), fulltime duration (*r* = -0.20, *p* < 0.05), and work experience (*r* = -0.31, *p* < 0.05), correlated inversely with intention to leave. While hours spent on research per week (*r* = 0.18, *p* < 0.05) has a direct and significant correlation with intention to leave.

As shown in Table 2, the mean scores of all dimensions of work-life quality and satisfaction and intention to leave were higher than 2.73 (out of 6). At the dimension level, faculties were most satisfied of their “technology support” in work-life quality scale (3.99, SD = 0.86) and average score for overall satisfaction (4.90, SD = 0.89) was higher than the satisfaction for the “advising and course workload” and “benefits and security”.

**Table 2:** The mean score of work-life quality, satisfaction, and intention to leave among study subjects (*n* = 120)

Scale & dimensions	Mean (SD)	Minimum	Maximum
Work life quality scale	3.83 (.62)	2.31	5.44
Administrative support	3.93 (.88)	1.25	6
Professional development	3.56 (.66)	2	5.67
Technology support	3.99 (.86)	1.33	6
Satisfaction scale	4.03 (.41)	2.5	4.83
Advising and course workload	3.94 (.54)	2	5
Benefits and security	3.27 (.54)	2	4.5
Overall satisfaction	4.90 (.89)	1	6
Intention to leave scale	2.73 (1.33)	1	6
How likely are you to leave your current position?	2.86 (1.46)	1	6
How likely are you leave their current institution?	2.80 (1.43)	1	6
How likely are you leave the teaching profession?	2.60 (1.38)	1	6
How likely are you leave higher education?	2.65 (1.31)	1	6

The least satisfaction among faculties was in the “benefits and security” dimension (3.27 ± 0.54). Also, in the scale of intention to leave the highest score among faculties was in statement of “what likely are you leave their current position?” (2.86 ± 1.46).

There was a statistically significant inverse relationship between the mean scores in all work-life quality and satisfaction dimensions and intention to leave, except for “advising and course workload” dimension (*p* < 0.05). Also, there was a statistically significant direct relationship between total scores of work life quality and satisfaction (*p* < 0.001) (Table 3).

**Table 3:** Correlations among subscales of work-life quality, satisfaction, and intention to leave among academics

Variables	X1	X2	X3	X4	X5	X6	X7	X8
Advising and course workload (X1)	1							
Benefits and security (X2)	.23^b^	1						
Overall satisfaction (X3)	0.15	-0.11	1					
Total Satisfaction (X4)	.64^b^	.45^b^	.73^b^	1				
Administrative support (X5)	.26^b^	.37^b^	.24^b^	.45^b^	1			
Professional development (X6)	.23^b^	.38^b^	0.09	.34^b^	.43^b^	1		
Technology support (X7)	.33^b^	0.13	.28^b^	.40^b^	.37^b^	.36^b^	1	
Total work life quality (X8)	.36^b^	.37^b^	.28^b^	.52^b^	.80^b^	.73^b^	.77^b^	1
Intention to leave (X9)	-0.1	-.21^a^	-.26^b^	-.32^b^	-.21^a^	-.29^b^	-.20^a^	-.30^b^

^a^Correlation is significant at the 0.05 level

^b^Correlation is significant at the 0.01 level

The results of the univariable linear regression analysis indicate that all subscale scores of Work Life Quality, as well as the total score, exhibited a negative correlation with the intention to leave (all *P*-values < 0.05). Likewise, within the Satisfaction subscales, Benefits and Security, Overall Satisfaction, and their total scores displayed an inverse relationship with the intention to leave (all *P*-values < 0.05). Examining demographic and background variables, age, work experience, and full-time years demonstrated a negative association with the intention to leave (all *P*-values < 0.05). Conversely, the number of hours spent by faculty members on research exhibited a positive correlation with the intention to leave (*P*-value < 0.05). Additionally, when compared to nursing faculty members as the reference category, Medicine, Midwifery, and Allied Health Professions exhibited lower intention to leave (all *P*-values < 0.05) (Table 4).

**Table 4:** Uni- and multi-variable linear regression results of factors affecting intention to leave in faculty members

	Uni-variable				Multi-variable			
Predictors	B	95% CI Lower	95% CI Upper	*P*-value	Adjusted B	95% CI Lower	95% CI Upper	*P*-value
Work life quality								
Subscale1: Administrative support	-0.31	-0.58	-0.041	0.024	.062	-.247	.372	.691
Subscale2: Professional development	-0.582	-0.93	-0.235	0.001	-.132	-.555	.291	.537
Subscale3: Technology support	-0.313	-0.588	-0.038	0.026	-.189	-.524	.147	.267
Work life quality (total)	-0.63	-1.009	-0.263	0.001	–-	–-	–-	–-
Satisfaction								
Subscal1: Advising and course workload	-0.252	-0.697	0.193	0.264	.141	-.331	.613	.554
Subscal2: Benefits and security	-0.509	-0.948	-0.07	0.024	-.339	-.821	.144	.167
Subscal3: Overall satisfaction	-0.398	-0.66	-0.135	0.003	-.255	-.553	.044	.094
Satisfaction (total)	-1.033	-1.584	-0.483	< 0.001	–-	–-	–-	–-
Age (years)	-0.051	-0.079	-0.023	0.001	.005	-.058	.068	.867
Work experience	-0.047	-0.073	-0.02	0.001	-.097	-.181	-.012	.025
Fulltime (years)	-0.032	-0.063	-0.002	0.038	.074	-.007	.154	.073
Research (hours in week)	0.017	0	0.034	0.048	.013	-.004	.031	.127
Discipline	-	-	-	-				
Nursing (reference)								
Medicine	-0.897	-1.559	-0.235	0.008	-.658	-1.378	.063	.073
Midwifery	-1.547	-2.391	-0.703	< 0.001	-1.289	-2.232	-.346	.008
Allied health professions	-0.732	-1.39	-0.073	0.03	-.332	-1.058	.394	.367
Health management and medical information	-0.54	-1.486	0.406	0.261	-.655	-1.645	.335	.192

Multivariable: R^2^ = 0.28, *P*-value = 0.003

–**-**Total scale score was not entered in the multivariable model to avoid multicollinearity

In the multivariable analysis, the relationship between work life quality, satisfaction subscales, and their total score with the intention to leave was found to be statistically non-significant (all *P*-values > 0.05). However, work experience and Discipline emerged as independent predictors of the intention to leave (both with *P*-value < 0.05). Specifically, work experience was negatively associated with the intention to leave, and each year of experience was linked to a 10% decrease in the intention to leave score points. Furthermore, faculty members in the midwifery discipline displayed approximately 1.3 points lower intention to leave scores compared to nursing faculty members (Table 4).

## Discussion

This study was conducted with the aim of investigating the work-life quality and satisfaction of faculty members of Urmia University of Medical Sciences and their relationship with intention to leave. The results revealed that the overall quality of work life was in an average level, with the highest scores observed in the "Technology Support" dimension. It must be acknowledged, in today's digital era, the Internet has emerged as a crucial tool for research development and enhancing the efficiency of academic staff members in universities [30]. Without utilizing the internet for various tasks, including education, research, and consultation responsibilities, academic staff members can encounter numerous challenges. Meanwhile, with the outbreak of the COVID-19 pandemic and the consequent shift to virtual classes, the necessary technological support has been provided to professors, ensuring their active participation in online teaching [31]. Previous studies have also reported similar findings, indicating an average level of work-life quality [32–35]. Furthermore, a meta-analysis of domestic articles conducted by Shakibaei (2015) showed that the average score of the quality of work life among academic staff members in higher education institutions is considered to be on a medium level [36]. However, studies conducted by Farrukhnejad (2012), Mirkamali and Thani (2011), and Noorshahi and Samiei (2023) reported an unfavorable level of quality of work life [37–39]. Another studies highlighted that faculty members experienced a lower quality of work life compared to other university employees, often citing unfavorable working conditions, lack of control and participation in decision-making, and low organizational commitment as contributing factors [40, 41]. Additionally, Bakhshi et al. found a direct relationship between the academic staff members' educational level and their perception of quality of work life [33]. It is important to note that the inconsistencies observed across different studies could potentially be attributed to variations in populations studied [40, 42] and the instruments utilized for assessment [43–45].

Based on the findings of this study, the average job satisfaction scores fell within the moderate range. Notably, the highest satisfaction scores were reported in the 'Overall satisfaction' dimension, while the lowest scores were observed in the 'Benefits and security' dimension. In terms of marital status and involvement in clinical teaching, married faculty members without clinical education responsibilities exhibited higher levels of satisfaction compared to their unmarried peers involved in clinical education. These findings align with previous studies which have also reported medium ranges of job satisfaction [32, 34, 37, 38], thereby corroborating the results of our present study. Noorshahi and Farastkhah's (2012) study identified various factors that contribute to faculty members' job satisfaction, including satisfaction with salaries and wages, the work environment, job security, job prestige and dignity, and facilities and resources. The study revealed that faculty members reported moderate to high satisfaction in terms of job prestige and dignity, whereas satisfaction with salaries and wages, the work environment, and job security were reported as moderate to low [30].

A positive work environment characterized by independence, role clarity, and community impact fosters higher job satisfaction, whereas dissatisfaction with salaries, weak leadership, and excessive pressure to produce scientific articles can lead to decreased job satisfaction. Job satisfaction is significantly linked to traditional academic values such as a focus on quality, inclusion in decision-making processes, unwavering commitment to work, and recognition of faculty members [40]. Additionally, faculty members' perception of organizational support enhanced job satisfaction [46]. In Moloantoa's study, it was observed that salary did not significantly impact job satisfaction, but dissatisfaction stemmed from insufficient benefits, inadequate support for teaching, learning, and research, lack of resources, and subpar university management [47]. In a study conducted by Ferron (2017) found that when nursing department managers actively supported academic professionals, recognized their efforts, and ensured fair work procedures, nurses' job satisfaction increased [48].

There is a statistically significant relationship between job satisfaction and the quality of work life [34, 49]. One of crucial aspect of the quality of work life is work-life balance [50]. Numerous studies worldwide have reported a positive association between job satisfaction, the quality of work life, and work-life Balance [51–58]. A study by Kim (2023) revealed that the high stress experienced by faculty members in Thailand mediates the relationship between high workload and job satisfaction [59].

The average score for the intention to leave was in the medium range. The item 'How likely are you to leave your current position?' scored the highest. Nursing faculty members exhibited a relatively higher tendency to consider leaving. The intention to leave was directly correlated with the number of research hours and inversely with work experience, full-time employment and age. Aboudahab's study on private universities in Egypt revealed that common factors contributing to the intention to leave included low talent management, high workload and anxiety, poor communication between faculty members and managers, lack of recognition and appreciation, and work-family imbalance [60]. Ferron (2017) found that intention to leave increased with aging among nursing faculty, as well as part-time employment. On the other hand, more work experience decreased the intention to leave and increased the desire to remain in nursing schools [48]. The low level of job satisfaction among faculty members is considered a warning sign, as it increases the likelihood of leaving a job if greater satisfaction is found elsewhere [60].

There was a statistically significant inverse relationship between the mean scores in all dimensions of satisfaction with work-life quality and the intention to leave, except for the "Advising and course workload" dimension. In the same line, other studies have also demonstrated a decrease in the quality of work life and job satisfaction leading to an intention to leave [24, 46, 57, 58, 60–66]. Job satisfaction plays a pivotal role in the retention of faculty members in universities [67]. In Rezaee’s study (2019) among Iranian doctors, a significant inverse relationship was found between the quality of work life and the intention to leave. When the quality of work life improves, it reduces the intention to leave and increases employee satisfaction [68]. Therefore, organizations can provide personal and social support to make employees feel valued and proud [66].

## Conclusion, implications, and recommendations

The findings of this study highlighted that the faculty members’ work-life quality and satisfaction, and the intention to leave were in an average level. There is a negative correlation between Work-Life Quality and Satisfaction subscales, along with demographic factors, and the intention to leave, while work experience and Discipline were significant independent predictors of intention to leave.

These results emphasize the need to prioritize and improve the conditions that foster job satisfaction in academia, as it plays a vital role in training the next generation and advancing education in universities. Of particular concern is the high intention to leave among nursing lecturers, which signifies the immense work pressure they face. Without proper support from nursing schools in terms of human resources, there is a risk of a decline in the nursing workforce due to an increasing number of faculty members leaving their positions. This could ultimately lead to a reduction in the quality of undergraduate nursing education in the long run. These findings offer valuable insights for academic institutions, highlighting the importance of fostering a supportive work environment and retaining faculty members. By addressing the factors influencing job satisfaction and intention to leave, institutions can enhance the overall satisfaction of their faculty members and promote longevity in their academic careers. Considering these results, it is recommended that future research delve into additional variables and interventions to further augment faculty satisfaction and mitigate the intention to leave within the academic setting. These efforts can contribute to the overall improvement of the academic environment, ensuring quality education and sustained academic excellence in the years to come. Considering the high rate of intention to leave among nursing faculty members, it is advisable to conduct qualitative studies to explore the nature of the quality of work life, job satisfaction, and the underlying causes of the intention to leave within this group. Additionally, experimental studies can be conducted to investigate the effects of organization-oriented interventions aimed at enhancing the quality of work life, job satisfaction, and reducing the intention to leave."

### Limitations

This study was conducted among the faculty members of Urmia University of Medical Sciences, and its results cannot be generalized to other universities. It is recommended to conduct similar studies in other medical science universities. Due to the relatively small sample size and the study being limited to one university, the generalizability of the current study is restricted. Thus, future studies with a larger and more diverse population are suggested.

## Data Availability

The datasets used and/or analysed the current study are available from the corresponding author upon reasonable request.

## Abbreviations

SD: Standard deviation
M: Mean
SPSS: Statistical Package for Social Sciences
